# Pharmacopœial Doses

**Published:** 1915-01-30

**Authors:** 


### PHARMACOPŒIAL DOSES.

Prior to the issue of the new Pharmacopœia various suggestions were advanced relative to the presentation of doses in the official volume. There were a few voices, and a few only, which advised that Pharmacopœial doses should be abolished *in toto*. Others urged that an attempt should be made to make the doses of similar preparations, as tinctures, extracts, etc., of uniform weight or measure, in order to spare the memory of the prescriber. A third suggestion was that doses should be quoted in metric numbers, so that students, from the outset of their career, should be encouraged to think and prescribe in terms of the decimal notation; while a fourth proposal argued that as the official doses, so long as they appeared in unqualified terms, seemed to limit the freedom of the physician, they should in every instance be qualified as "pharmaceutical doses." Now that the new edition has been launched we are able to judge with what measure of favour these several suggestions have been regarded by the authorities. The first two have been left severely alone, and with this decision we are in complete agreement. The third has been adopted, at least to this extent, that the official doses are quoted in the decimal notation as well as in terms of the imperial weights and measures. In reference to the fourth proposal, the Committee appears to have recognised the claim that official doses do involve some element of prejudice to individual judgment, though they have not placed in the text of their volume the individual qualification suggested. This recognition places a new responsibility on the dispenser, and indirectly calls for the co-operation of the physician.

In the preface to the official volume there is to be found the authoritative general statement which indicates the nature and extent of the jurisdiction exercised by the Pharmacopœial doses. This statement, in substance, announces that while such doses are "meant for general guidance" they are "not authoritatively enjoined"; and indicates that the medical practitioner must act on his own responsibility as to the doses of any therapeutic agents which he may administer. In other words, whilst a scheme of official doses has a certain general utility, it in no wise limits the freedom or minimises the responsibility of the physician. Its advantage is mainly in the protection it affords to the public against the risk of accidental error on the part of the prescriber. In the event of such error the dispenser, informed by the Pharmacopœia, is in a position to note the mistake and to ask the writer of the prescription to make the necessary correction.

The new edition carries still further the presentation of official doses as a means of communication, in the public interest, between pharmacist and physician. It reaffirms what was stated in former editions as to the general purposes served by such doses and as to the freedom and independence of the prescriber. But it now adds "where, however, an unusually large dose appears to be prescribed, it is the duty of the pharmacist or dispenser to satisfy himself that the prescriber's intention has been correctly interpreted," though "correctly expressed" would surely have been a more accurate phrase. In taking this position the Pharmacopœia places on the dispenser a statutory obligation where previously there was merely a bond of courtesy or custom. It will be necessary in future for anyone engaged in the dispensing of physicians' prescriptions to be sure that the doses ordered in each instance are not in excess of those quoted in the Pharmacopœia. As such a regulation is a further safeguard for the public it is in full harmony with the main purpose of the Pharmacopœia, and it merits the co-operation and approval of all good citizens.

It is necessary that the medical profession shall take note of this recent development. Unless some other plan is devised the dispenser must refer every such prescription to the prescriber for his express warrant and authority. The avoidance of the delay and annoyance which would thus be caused is easily accomplished if, when any unusually large dose of a remedy is ordered, the dose is specially initialled by the physician on the margin of the prescription. The Pharmaceutical Society has issued a proposal to this effect, and we have pleasure in supporting so reasonable an appeal.

